# A novel mutation in C5L2 gene was associated with hyperlipidemia and retinitis pigmentosa in a Chinese family

**DOI:** 10.1186/1476-511X-13-75

**Published:** 2014-05-06

**Authors:** Ling-hui Qu, Xin Jin, Liang-mao Li, Shi-ying Li, Han-ping Xie

**Affiliations:** 1 Southwest Eye Hospital, Southwest Hospital, Third Military Medical University, 30 Gaotanyan Road, Chongqing 400038, China; 2 Department of Ophthalmology, Chinese PLA General Hospital, Beijing 100853, China; 3 Department of Ophthalmology, No. 181 Hospital of Guilin, Guilin 541002, China

**Keywords:** C5L2, Mutation, Retinitis pigmentosa

## Abstract

**Background:**

Previous studies indicated that hyperlipidemia was associated with retinitis pigmentosa (RP). We aimed to identify the mutations in the C5L2 gene which was reported to be associated with hyperlipidemia in a Chinese family with (RP).

**Methods:**

The Proband from the family was screened for mutations in the *C5L2* gene that was known to cause hyperlipidemia. Cosegregation analysis was performed in the available family members. Linkage analysis was performed for one missense mutation to calculate the likelihood of its pathogenicity. One hundred and fifty unrelated, healthy Chinese subjects were screened to exclude nonpathogenic polymorphisms.

**Results:**

By direct sequencing method, we identified a novel mutation (Thr196Asn) in C5L2 gene. In this family, each affected family members with RP showed a heterozygous mutation in the *C5L2* gene. And all the carriers with heterozygous mutation have increased serum lipid levels in this family.

**Conclusions:**

The present study has extended the mutation spectrum of *C5L2,* and Thr196Asn mutations in *C5L2* were associated with RP and serum lipid levels.

## Introduction

Retinitis pigmentosa (RP) is a hereditary retinal degeneration of unknown etiology, resulting in progressive night blindness, loss of peripheral vision, abnormal retinal pigmentation and reduced electroretinographic response [1]. Nonsyndromic RP is the most common inherited form of severe retinal degeneration, with a prevalence of approximately 1/4,000 RP cases worldwide [1]. Nonsyndromic cases can be inherited as an autosomal-dominant (20%–25%), autosomal-recessive (15%–20%), X-linked recessive (10%–15%), or sporadic/simplex (30%) trait [2].

Although there are more than 55 genes have been reported to be associated with RP, the mechanism of RP remains unclear. The previous studies suggested that lipid metabolism was abnormal in patients with RP [3,4]. And Fujita et al. reported an association between a missense coding region polymorphism Asn985Tyr in the retinitis pigmentosa 1 gene (RP1), a causal gene for RP, and plasma triglyceride (TG) levels in 332 adult Japanese [5]. These evidences suggested that abnormal metabolism of lipids may be associated with the RP patients. C5L2 is a G protein-coupled receptor (GPCR) and was demonstrated to be a functional receptor of acylation-stimulating protein (ASP) [6-8]. ASP is also known as C3a des-Arg, a stimulator of TG synthesis [9-12] or glucose transport [13,14]. Previous study suggested that genetic mutation of C5L2 was associated with hyperlipidemia [15,16]. Therefore, we hypotheses that the mutation in C5L2 gene is associated with risk for RP.

In the present study, we aimed to identify novel mutations in C5L2 gene in a RP family and to analyze the relation between this mutation and RP.

## Methods

### Recruitment of subjects

**Figure 1:**
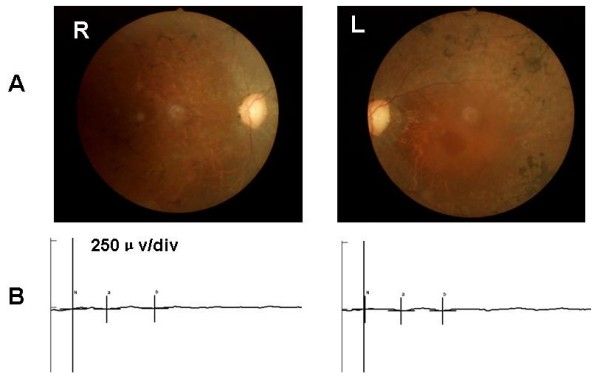
**Morphological findings. A**. Fundus pictures of the right (R) and left eye (L) of the index patient showing pallor optic discs, attenuated vessels, and characteristic pigment changes in the mid-periphery. **B**. ERG testing showed extinguished rod response.

All participants were identified at Southwest Eye Hospital, the General Hospital of Chinese People’s Liberation Army. The diagnosis of RP was based on the presence of night blindness, typical fundus findings (characteristic retinal pigmentation, vessel attenuation, and various degrees of retinal atrophy), the severe loss of peripheral visual field, and abnormal electroretinogram (ERG) findings (dramatic diminution in amplitudes or the complete absence of response). Figure 1(A) showed the fundus pictures of the right (R) and left eye (L) of the index patient. ERG testing showed non-recordable responses (Figure 1B). This study was approved by the Institutional Review Board of Southwest Hospital, the General Hospital of Chinese People’s Liberation Army and adhered to the tenets of the Declaration of Helsinki and the Guidance on Sample Collection of Human Genetic Diseases by the Ministry of Public Health of China.

### Clinical evaluations

For each patient, a full medical and family history was taken and an ophthalmological examination was performed. Each underwent a standard ophthalmic examination: best correct visual acuity according to Snellen charts, slit-lamp biomicroscopy, dilated indirect ophthalmoscopy, fundus photography if possible, and visual field tests (Octopus; Interzeag, Schlieren, Switzerland). Retinal structure was examined by using optical coherence tomography (OCT; Topcon, Tokyo, Japan). ERGs were performed (RetiPort ERG system; Roland Consult, Wiesbaden, Germany) using corneal “ERGjet” contact lens electrodes. The ERG protocol complied with the standards published by the International Society for Clinical Electrophysiology of Vision.

### Biochemical analysis

Serum concentrations of total cholesterol (TC), Triglycerides (TG), glucose, high-density lipoprotein cholesterol (HDL-C), Low-density lipoprotein cholesterol (LDL-C) were measured using standard methods.

### Genetic studies

Genomic DNA was extracted from the peripheral white blood cells. We used 2ml blood which collected in EDTA vacutainer tubes. Genomic DNA was isolated from the peripheral leukocytes by using a QIAamp DNA Blood Midi Kit (Qiagen, Hilden, Germany) according to the manufacturer’s protocol. All 2 coding exons of the C5L2 gene were sequenced. Sequence information for use as a reference template was obtained from the Ensembl Genome Browser (Human, number ENSG00000134830). Sequencing primers were described by Zheng et al. [15,16]. The sense primer was 5′AAGATGCCACTTCTA ACAACA3′ and the antisense primer was 5′GTTGAATGAAGGAAGGAATAA3′. The PCR procedure was described previously [16]. Briefly, the polymerase chain reaction (PCR) was undertaken with 50 ng of genomic DNA in a 20 μL reaction containing 10 μL of Power Mix (Beijing Biotech, Beijing, China), 9.5 μL of distilled water, and 0.2 mM of each forward and reverse primer. A GeneAmp 9700 thermal cycler (Applied Biosystems, Foster City, CA, USA) was used for PCR amplification. An initial denaturation step at 95°C for 5 min, 40 cycles of 95°C for 30 s, 56°C for 30 s, and 72°C for 1 min was followed by a final extension step of 72°C for 10 min. A 1615-base pair (bp) product was amplified was purified using ExoSAP-IT (Amersham Biosciences) according to manufacturer’s instructions before it was used as a template for sequencing. Sequencing reactions were undertaken by BGI-Beijing (Beijing, China; http://www.genomics.cn).

### Statistical analysis

Data were analyzed using SPSS software 13.0. Statistical significance was accepted at *p* < 0.05. Values were expressed as mean ± SD for serum concentrations of TC, TG, HDL-C and LDL-C. Student’s t-test was performed to compare serum lipids levels between mutation carriers and wild carriers.

## Results

### Mutation analysis

Upon complete sequence analysis of the coding regions of *C5L2*, three mutations were detected in four (RP patients) out of 16 family members, including one novel missense mutation (Thr196Asn) and two previously reported missense mutation (Pro233Leu, and rs36046934). The missense mutation c.587C>A in exon 1 resulted in a substitution of asparagine for threonine at codon 196 (Thr196Asn). This novel mutation (Thr196Asn) has not been found in 150 unrelated healthy subjects.

### The relation between novel mutation and RP

**Figure 2:**
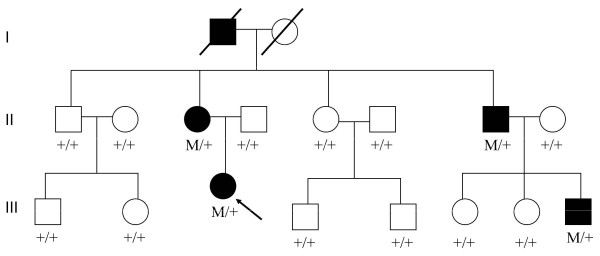
**Pedigree of the Chinese family with retinitis pigmentosa associated with mutations in the C5L2 gene.** Genotypes are shown beneath the symbols. Affected individuals are represented by black symbols, unaffected ones by unfilled; squares signify males, circles females. Arrows mark the index patients. M refers to the mutant allele, and + means normal allele.

**Figure 3:**
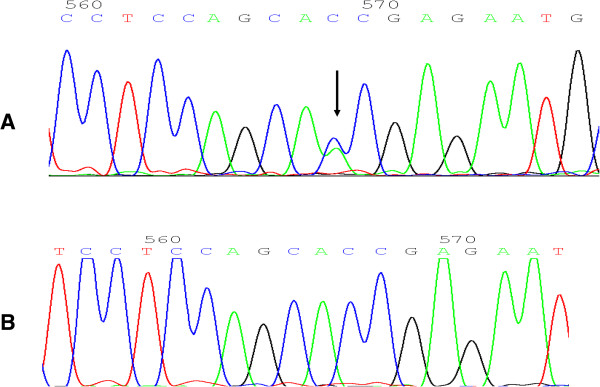
**The DNA sequence around the mutation site. A**: Mutation sequence; **B**: wild sequence.

In this family, we found all these four patients with RP were carriers of 196Asn allele, but the else 10 unaffected members were not carriers with 196Asn allele (Figures 2 and 3).

### The relation between novel mutation and lipids level

**Figure 4:**
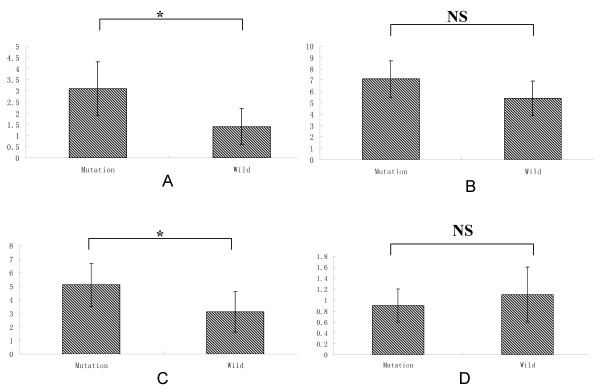
**Comparation between mutation carrier and wild carrier in serum lipids levels. A**: TG; **B**: TC; **C**: LDL-C; **D**: HDL-C

In this family, we also found the carriers with 196Asn allele have higher levels of TG (2.9 ± 1.3 mmol/L) and LDL-C (5.1 ± 1.4) (*p <* 0.05). However, the subjects without 196Asn allele have lower levels of serum lipids (TG, 1.6 ± 0.4; LDL-C, 3.3 ± 1.1) (*p <* 0.05) (Figure 4).

## Discussion

In the present study, we identified a novel missense mutation (Thr196Asn) in a RP family, and found this variant affects the risk for RP and hypertriglyceridemia. This is the first study to identify a novel mutation and analyze the relation between this mutation and RP.

The foundation for human studies examining putative causative genes that may be involved in RP is based on a candidate gene approach. A few studies into the genetic polymorphisms of the C5L2 receptor located on chromosome 19q13 (the region identified to be associated with familial combined hyperlipidemia and the pre-diabetic state by genome-wide scan studies) have been completed [17,18]. Familial combined hyperlipidemia is considered to be the most frequent lipoprotein disorder in RP [3-5]. Therefore, the C5L2 gene is thought to be a candidate gene for RP.

In the present study, we identified three missense mutations in a family with RP. The Pro233Leu mutation was found by Zheng et al. in coronary artery disease (CAD) patient. Zheng et al. found the Pro233Leu variant was associated with CAD and Type 2 diabetes. The other variant- rs36046934 have been recorded in the NCBI database (http://www.ncbi.nlm.nih.gov/SNP). Therefore, in the present study, we did not analyze the relation between these two variants and RP. As for the mutation Thr196Asn, we did not find in 150 unrelated healthy subjects, which indicated that this mutation is a rare variant. In this RP family, all these four patients with RP have this mutation and the other eight members without RP have not this mutation, which suggested that the Pro233Leu variant is a causative mutation for RP in this family.

In addition, we also found the Pro233Leu variant was associated with increased TG and LDL-C levels in this family. However, the mechanisms may link to the RP and abnormal lipid level resulting from the C5L2 mutation is unclear and is worth exploring in the future.

## Conclusion

In conclusion, the C5L2 may be a causative gene for RP with hyperlipidemia.

## Competing interests

The authors declared no competing interests exist.

## Authors’ contributions

LHQ and XJ carried out the molecular genetic studies and drafted the manuscript. QLH and LML carried out the genotyping. ZQY, HPX and SYL participated in the design of the study and performed the statistical analysis. All authors read and approved the final manuscript.

## Authors’ information

Ling-hui Qu and Xin Jin co-first authors.
