# Correction: A novel mutation in C5L2 gene was associated with hyperlipidemia and retinitis pigmentosa in a Chinese family

**DOI:** 10.1186/1476-511X-13-110

**Published:** 2014-07-08

**Authors:** Ling-hui Qu, Xin Jin, Liang-mao Li, Shi-ying Li, Zheng-qin Yin, Han-ping Xie

**Affiliations:** 1

## Correction

After publication of this work [1], we noted that we inadvertently failed to include the complete list of all coauthors. The full list of authors has now been added
